# Exploring FAST Technique for Diffusion Bonding of Tungsten to EUROFERE97 in DEMO First Wall

**DOI:** 10.3390/ma17112624

**Published:** 2024-05-29

**Authors:** María Sánchez, Javier de Prado, Ignacio Izaguirre, Andrei Galatanu, Alejandro Ureña

**Affiliations:** 1 Materials Science and Engineering Area, Escuela Superior de Ciencias Experimentales y Tecnología, Universidad Rey Juan Carlos, C/Tulipán s/n, 28933 Móstoles, Spain; ignacio.izaguirre@urjc.es (I.I.); alejandro.urena@urjc.es (A.U.); 2 Instituto de Investigación de Tecnologías Para la Sostenibilidad, Universidad Rey Juan Carlos, C/Tulipán s/n, 28933 Móstoles, Spain; 3 National Institute of Materials Physics, Atomistilor 405 A, 077125 Magurele, Romania; gala@infim.ro

**Keywords:** tungsten, EUROFERE97, copper interlayer, diffusion bonding

## Abstract

The European Fusion Reactor (DEMO, Demonstration Power Plant) relies significantly on joining technologies in its design. Current research within the EUROfusion framework focuses on developing materials for the first wall and divertor applications, emphasizing the need for suitable joining processes, particularly for tungsten. The electric field-assisted sintering technique (FAST) emerges as a promising alternative due to its high current density, enabling rapid heating and cooling rates for fast sintering or joining. In this study, FAST was employed to join tungsten and EUROFERE97 steel, the chosen materials for the first wall, using 50-µm-thick Cu foils as interlayers. Three distinct joining conditions were tested at 980 °C for 2, 5, and 9 min at 41.97 MPa to optimize joint properties and assess FAST parameters influence. Hardness measurements revealed values around 450 HV_0.1_ for tungsten, 100 HV_0.1_ for copper, and 390 HV_0.1_ for EUROFER97 under all joining conditions. Increasing bonding time improved joint continuity along the EUROFER97/Cu and W/Cu interfaces. Notably, the 5 min bonding time resulted in the highest shear strength, while the 9 min sample exhibited reduced strength, possibly due to Kirkendall porosity accumulation at the EUROFER97/Cu interface. This porosity facilitated crack initiation and propagation, diminishing interfacial adhesion properties.

## 1. Introduction

The imminent deployment of future fusion power plants brings to the forefront the critical challenges posed by their high-power density and the extreme conditions prevailing within the vessel, characterized by intense irradiation, physical and chemical erosion, and sputtering. Addressing these challenges necessitates the development of advanced materials. Tungsten emerges as the primary candidate for providing armor to the first wall and divertor components, requiring effective integration with structural steel (EUROFER97) to meet stringent mechanical and metallurgical specifications. Therefore, the joining technology between both materials needs to be investigated. Tungsten, as a refractory metal with the highest melting point among all metallic materials, presents inherent physical and chemical properties for this task (high melting point, sputtering resistance, and thermal conductivity [1,2,3]). However, the high corrosion rate under air atmosphere at high temperatures has limited its application in the industry. As a result, its joinability has not been widely explored. The same properties that make this material adequate for this task also limit the available joining technologies, and an important effort should be made by the scientific community to address this issue.

Different techniques have been studied to join W to EUROFER97, including diffusion bonding (DB) processes [4,5], laser welding [6], and brazing [7,8]. However, the large differences in the thermal expansion coefficients (CTEs) make the bonding between W and EUROFER97 a big challenge [9,10]. Conventional methods have different limitations [11,12,13]. As an illustration, traditional diffusion bonding necessitates high temperatures and extended bonding times, making it a time- and energy-intensive process. Similarly, brazing involves the use of a filler metal with a lower melting point than the base materials, potentially compromising the mechanical properties of the joint under elevated temperatures and falling short of meeting application requirements.

In contrast, the electric field-assisted sintering technique (FAST), also known as spark plasma sintering (SPS), presents a novel approach for the swift consolidation of powders. This technique employs a robust, pulsed DC flow through powders or dies to generate joule heat, offering distinct advantages such as rapid heating rates, shortened sintering times, and low energy consumption [14,15,16]. The fusion of diffusion bonding with FAST technology emerges as a promising strategy for bonding tungsten with EUROFER97. Remarkably, the electrical current utilized in the FAST process promotes the migration of metallic and refractory ions at the joining interface, positively influencing the interfacial bonding strength of the joints [17].

Various researchers have explored the application of the FAST technique in joining materials, similar to those investigated in this study. For instance, D. G. Liu et al. [18] investigated the joint between an oxide dispersion-strengthened tungsten-based material and TZM, achieving optimal conditions at 1500 °C and obtaining a remarkable tensile strength of 485 MPa. In another study, Galatanu et al. [19] delved into a multi-layered composite between copper and tungsten, synthesized through a FAST-joining route. Notably, in heterogeneous joints, the Joule effect causes the material with the higher electrical resistivity to be heated more than the better electrical conductor. In the case of W–Cu laminates, tungsten is more heated than copper, and heat flows from tungsten towards copper at the interface. Structural, thermophysical properties, as well as tensile and Charpy impact tests conducted in this investigation, underscored that W–Cu laminates produced via FAST processing are comparable to those obtained through diffusion bonding. M. Naderi et al. [20] conducted investigations on the interaction between steel and copper, characterizing the microstructures and mechanical properties of Cu-AISI4140 steel solid-state joints created through spark plasma welding. Their examination included a comparative analysis of the process with and without a mold, revealing a notable reduction in unjoined areas and the formation of micropores confined at the joining interface. This modification contributed to an enhancement in the joining strength, escalating from 42 MPa to 90 MPa. 

The FAST technique has also been used to explore functionally gradient material (FGM) fabrication. A. Pasha et al. fabricated a multilayer material based on Cr, Ti, Fe, Ni, Co, and Cu elements via Spark Plasma Sintering. The proportion of elements of each layer was gradually modified to mitigate the stress generated during the joining process of the components [21,22]. Other works explored the formation of grade interlayers constituted by seven layers of W/Cu functionally graded material (100 W, 80W-20Cu, 60W-40Cu, 50W-50Cu, 40W-60Cu, 20W-80Cu, 100Cu, by wt.%) by a spark plasma sintering process (SPS). The results indicated the consecution of fine microstructure within each layer with good interface bonding [23,24].

The current study introduces varied conditions for diffusion bonding aimed at joining tungsten to EUROFER97 for the first wall of DEMO. The diffusion-bonded joints underwent comprehensive characterization, employing scanning electron microscopy for microstructural analysis and microhardness, as well as shear tests for mechanical assessment.

## 2. Materials and Methods

### 2.1. Materials

Pure tungsten and EUROFER97 were the base materials. W was supplied by Plansee (>99.97 wt.% purity, Breitenwang, Austria). EUROFER97 had a standard composition and microstructure with the following nominal chemical composition (wt.%): 0.11 C, 8.7 Cr, 1.0 W, 0.1 Ta, 0.19 V, 0.44 Mn and 0.004 S [25]. The pure Cu interlayer was supplied by Lucas-Milhaupt (with the commercial name *CDA 102K*, Cudahy, WI, USA, EEUU) and had a thickness of 50 µm. The interlayer had a composition of copper 99.95% Min, Oxygen 0.0010% Max and Other Elements (Total) 0.05% Max.

### 2.2. Joining by Field-Assisted Sintering Technique

The joining of W-EUROFER97 specimens was conducted using an FCT SPS D50 (Spark Plasma Sintering) equipment produced by *FCT Systeme GmbH* from Rauenstein, Germany. As the equipment allows only for integer values of force, an approach to achieve finer pressure tuning involved joining four samples simultaneously for each condition. The samples comprised blocks of W (6 × 6 × 5) mm, Cu foil (6 × 6 × 0.05) mm, and blocks of EUROFERE97 (6 × 6 × 5) mm. Prior to assembly, all pieces underwent thorough cleaning with 99.9% ethanol.

To facilitate alignment in the molds, a specialized tool was created and positioned between the pistons. An initial force of 2 kN was applied at room temperature to secure the pieces together. Subsequently, the tool was removed, and the die was raised to close the mold. The joining process unfolded within a low Ar atmosphere (<1 Pa), achieved through three vacuum/Ar-purging sequences.

The direct current pulses applied to heat the samples had a duration of 3 ms, with a 3 ms pause between pulses. The joining cycle encompassed a cold pressing step, reaching approximately 27 MPa, followed by a controlled heating phase, at a constant pressure, up to 450 °C, progressing at a rate of 100 °C/min. Simultaneously, the temperature increased at a rate of 40 °C/min up to 900 °C, accompanied by an elevation of pressure to 41.67 MPa. The final heating stage at this pressure reached up to 980 °C. Three dwell times were implemented: 2, 5, and 9 min. The selected bonding temperature ensures the austenitization of the steel during the bonding process (A_1c_ = 890 °C [25]), which will allow us to recover the hardness of the steel by applying a subsequent post-bonding heat treatment. The selected bonding pressure was set from 27 to 45 MPa, according to the literature review, where some works indicated optimal initial pressures around 25 MPa [19]. On the other side, as shown by Naderi et al. in Ref [20], for Cu steel joints, a pressure of 40 MPa achives enough plastic deformation, at this bonding temperature, to enhance the metallic contact. However, our FAST equipment allows only for force variation in 1 kN steps from 2 kN to 50 kN. The closest value available was 41.67 MPa, taking into account the joined surface area.

**Figure 1:**
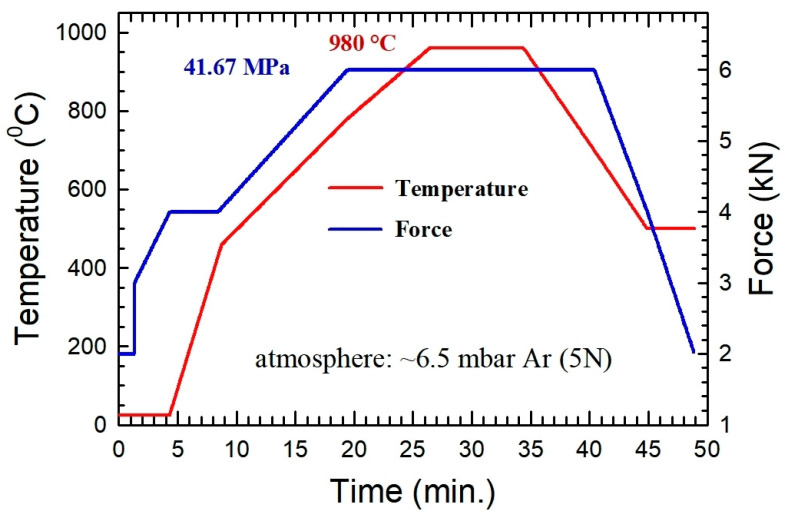
Scheme of the joining process conditions for the 9 min sample.

The cooling process was executed at a rate of 50 °C/min. Initially, there was a stepwise descent to 700 °C, maintaining constant pressure, followed by a gradual reduction in both temperature and pressure. Figure 1 shows a scheme of the joining process conditions for the 9 min sample.

### 2.3. Characterization Techniques

The microstructural analysis of diffusion-bonded sample cross-sections was conducted using scanning electron microscopy (SEM, *S3400 Hitachi*, Tokyo, Japan) equipped with energy dispersive X-ray analysis (EDX). Metallographic preparation of the samples followed the standard polishing technique. Mechanical properties of the joints were assessed through Vickers microhardness and shear tests. Microhardness profiles across W-EUROFER97 joints were generated using an *MHV-2 SHIMADZU* (Tokyo, Japan) apparatus. A 100 g load was applied for 15 s, with three measurements obtained at each position. Distances between neighboring indentations exceeded three times the residual imprint sizes.

Shear strength values were determined using a shear fixture positioned between compression platens in a universal testing machine (*Zwick Z100,* Ulm, Germany) at a speed of 1 mm/min. The fixture was specifically designed to align the shear force with the bonding interface, maintaining the sample position so pure shear forces could be applied. To ensure accuracy, three samples from each condition underwent measurements.

## 3. Results

### 3.1. Microstructural Analysis

**Figure 2:**
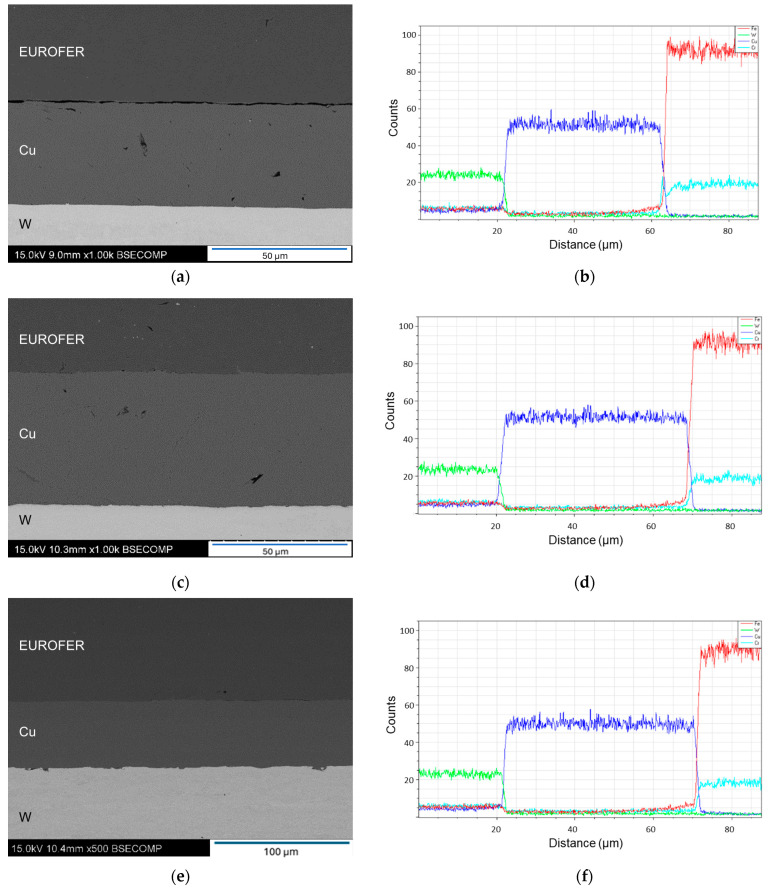
SEM micrographs of the W-EUROFER97 diffusion bonded joints at different conditions: (**a**) 2 min, (**c**) 5 min, and (**e**) 9 min. (**b**,**d**,**f**) Elemental diffusion distribution curves (Fe in red, Cr in blue, Cu in cyan and W in green) obtained from line scan analysis of EDX across the different joints corresponding to joining conditions (**a**), (**c**), and (**e**), respectively.

The microstructures of the three bonded joints under diffusion bonding (DB) conditions (980 °C for 2, 5, and 9 min at 41.97 MPa) are illustrated in Figure 2. Joints bonded with the shortest duration show the consecution of a continuous interface along the W/Cu region, with only isolated bonding zones evident at the EUROFER97/Cu interface (Figure 2a). These interface discontinuities suggested that the mechanisms of creep and interfacial diffusion were not conducive at the EUROFER97/Cu interface. Microstructural analysis revealed the presence of cracks along the interface, likely formed during the cooling stage. The dissimilarity in thermal expansion coefficients between Fe and Cu, combined with the insufficient interfacial strength, may contribute to this phenomenon. This fact is commonly observed when materials with a different coefficient of thermal expansion are joined and subjected to thermal processes. The different volume expansion of both materials generates internal stresses, which could lead to a crack nucleation or complete fracture of the joint/component in cases where the presence of low toughness phases or materials could not relieve the residual stress [21,22,26]. In this specific case, the lower adhesion properties of the EUROFER97/Cu interface promote the nucleation and propagation of the cracks following this interface.

Figure 2b,d,e reveal continuous and smooth variations in the Fe and Cu profiles under all three bonding conditions, suggesting interdiffusion between the materials across the interface. This interdiffusion predominantly occurred along grain boundaries, extending over a few microns. Furthermore, copper diffuses into the bulk grain, a phenomenon that will be elaborated upon in the subsequent microstructural discussion. Particularly, the W/Cu interfaces undergo a similar process; however, in this instance, the dissolution of W, and its subsequent precipitation, plays a pivotal role in shaping the observed smooth interface.

EDS punctual analyses carried out on both sides of both interfaces at distances of 0.5 microns form the interface line, indicating compositions of 89Fe-8Cr-3Cu at.% and 87Cu-12Fe-1Cr at.% at the EUROFER97 and Cu filler sides for the EUROFER97–Cu interface, respectively. In the case of the W–Cu interface, compositions of 75Cu-25W at.% and 91W-9Cu at.% for the Cu and W side, respectively, were detected. This information confirms the interdiffusion process of the elements that constitute the joint. 

The extension of bonding time, specifically up to 5 and 9 min, significantly promoted the bonding process, effectively preventing the occurrence of cracking at the EUROFER97/Cu interface, as illustrated in Figure 2c and Figure 2e, respectively. The analysis of the W/Cu interface demonstrated the successful attainment of complete metallic continuity under all three FAST conditions. Despite the inherent low-temperature immiscibility of W and Cu, coupled with the refractory nature of W, a distinct interface emerged from the microstructural perspective. Remarkably, no metallurgical interactions, such as interdiffusion or the formation of third phases, were observed. Nonetheless, the plastic deformation of the copper filler at the bonding temperature facilitated the crucial establishment of intimate contact, raising metallic continuity and promoting the formation of metallic bonds.

In addition, the lack of miscibility can be overcome by increasing the temperature up to temperatures close to the copper melting point because diffusion paths could be created. J. Zhang et al. [27] successfully constructed a metallurgical bonding interface between W and Cu through diffusion, giving rise to W/Cu joints characterized by remarkable strength.

A deep examination of both interfaces at higher magnification was conducted to identify any potential welding defects or discontinuities. While these areas hold significance in conventional welding techniques, their importance is further emphasized in the FAST technique. In these zones, the initial contact area is low, leading to an elevated current flow and a substantial increase in temperature due to the Joule effect. The scrutiny was concentrated on the samples subjected to 5- and 9-min bonding times, as they exhibited superior weldability.

**Figure 3:**
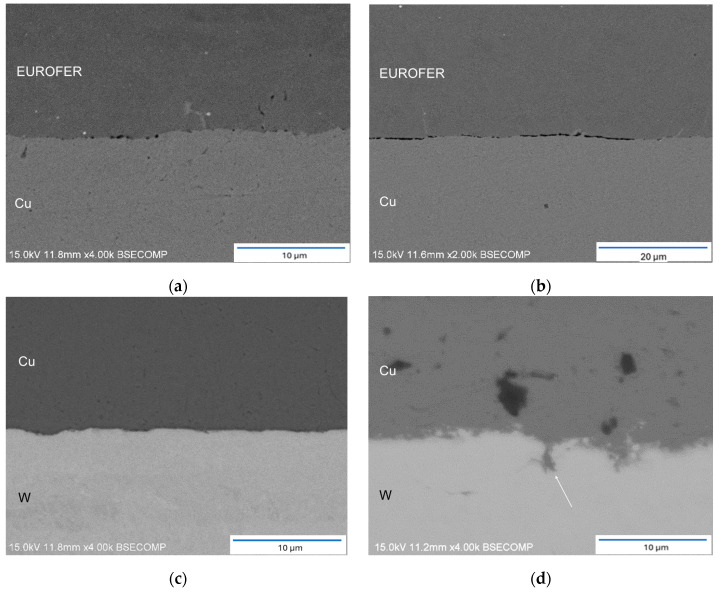
Detail of EUROFERE97/Cu interface of joints bonded at: (**a**) 5 min and (**b**) 9 min. Detail of W/Cu interface of joints bonded at: (**c**) 5 min and (**d**) 9 min.

Figure 3a,b depict images corresponding to the EUROFER97/Cu interface for the 5 min and 9 min DB durations, respectively. In particular, microporosity is evident in both samples, with localized occurrences in the 5 min sample and a more abundant presence in the 9 min sample, extending along the interface. In certain regions of the lengthiest DB duration, this porosity becomes continuous. The observed location and geometry of the porosity suggest the formation of Kirkendall porosity during the DB process. This phenomenon arises when a significant disparity in the diffusion coefficients of the involved elements within the parent crystalline structure is established. According to the literature, at a temperature of 980 °C, the diffusion coefficient of copper in gamma iron is reported to be 7.63 × 10^−13^ cm^2^/s [28], whereas the diffusion coefficient of iron in copper at the same temperature is notably higher at 1.26 × 10^−9^ cm^2^/s [29]. Consequently, the accumulation of vacancies at the interface leads to porosity along the interface line, a process known as the Kirkendall effect.

The analysis of the W/Cu interface revealed local interaction between tungsten and copper, particularly in the 9 min sample (Figure 3d), where W diffuses and, during cooling, precipitates under these conditions within the Cu matrix. Conversely, a comparatively lower level of interaction was observed in the 5 min sample, attributed to its shorter exposure to the bonding temperature (Figure 3c). In the case of the extended DB duration, copper penetration along the W grain boundary was evident (indicated by the white arrow in Figure 3d). The inherent nature of FAST technology may elucidate this phenomenon. In the initial stages of the process, the contact area between W and copper is notably limited, causing the current to concentrate in these regions. This localized concentration of current could result in a temperature increase several hundred degrees higher than the bulk material, potentially leading to the local melting of the copper filler or an enhanced diffusion coefficient in the grain boundaries, facilitating copper migration. In addition, close to the melting point of copper, W could penetrate through a diffusion mechanism through copper grain boundaries, leading to partially solubilized W in the closest area of the joint interface. This W precipitates when the solubility decreases during the cooling stage of the process.

In general aspects, the obtained microstructure is similar to that obtained with other solid-state diffusion techniques. For example, E. Sal et al. studied the use of the HIP technique to join W to EURFOER97. They did not report extensive diffusion phenomena obtaining well-defined interfaces between the filler and the base materials. However, the intrinsic characteristic of this technique implies the application of longer joining times [30,31]. On the other hand, the use of liquid state joining techniques usually reports higher interactions at the bonding interfaces. For example, I. Izaguirre et al. reported copper penetration of the EUROFER97 grain boundary at the ERUFOER97/braze interface and the formation of a reaction layer at the W–braze interface, giving rise to a more complex microstructure [32,33].

### 3.2. Mechanical Characterization

**Figure 4:**
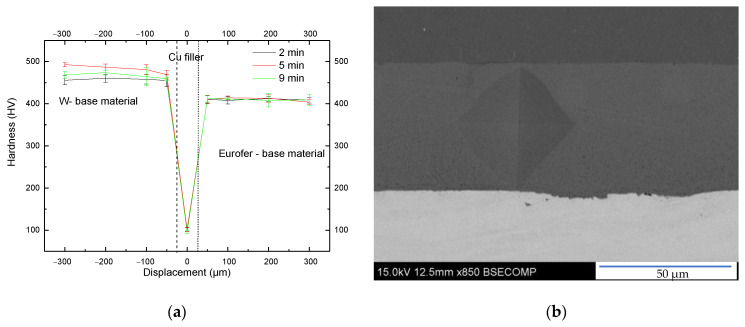
(**a**) Vickers microhardness profiles of W-EUROFER97 joints under various evaluated conditions. (**b**) SEM image if the Vickers imprint carried out at the copper filler.

Figure 4 illustrates the microhardness profiles extending from the tungsten base material to the EUROFER97 region across the joint. The profiles displayed remarkable similarities, indicating that the bonding time did not exert any discernible influence on the measured microhardness. The individual hardness values for tungsten, copper, and EUROFER97 were approximately 450, 100, and 390 HV_0.1_, respectively. Tungsten hardness remained unaffected by the bonding process, maintaining its initial conditions. However, EUROFER97 exhibited an increased hardness compared to its as-received state (210 HV_0.1_ [25]). The diffusion bonding temperature of 980 °C induced a hardness similar to that of EUROFER97 after undergoing austenization treatment and martensitic transformation when cooling rates higher than 5 °C/min is applied (~390 HV_0.1_ [34]). In this work the utilized high cooling rate was effective in producing a non-equilibrium transformation of austenite into martensite, thereby elevating the material hardness. To restore EUROFER97 initial hardness, the joint necessitates a tempering treatment at 760 °C for 90 min, facilitating the recovery of the tempered martensite characteristic of this steel, restoring the dislocation density generated during the martensite formation and previous thermomechanical treatment, and producing the precipitation of the carbides that provide the precipitation hardening effect typical of this high temperature tempering process, allowing the application of the steel in high temperature conditions [35].

The analysis of the microhardness imprints carried out at the Cu filler did not reveal the presence of crack nucleation or propagation in the joint area (Figure 4b). Although this test is characterized by the application of forces in a reduce area and the nature of the Vickers imprints generated stress concentration points close to both interfaces (vortex of the imprint), both filler and interfaces have accommodated the stress without crack formation, which positively inform us about the toughness of the joint area and adhesion properties of the interfaces. 

**Figure 5:**
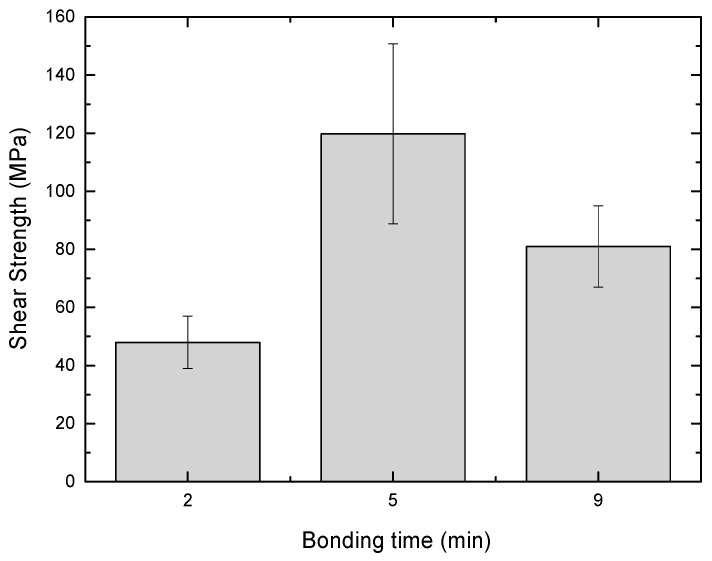
Shear strength of the W-EUROFER97 joints under various evaluated conditions.

The shear strengths of the diffusion bonded joints were 48 ± 9 MPa, 120 ± 31 MPa, and 81 ± 14 MPa for samples bonded during 2, 5, and 9 min, respectively (Figure 5). Particularly, the joint bonded during the initial 2 min period exhibited lower strength, primarily attributed to the discontinuous EUROFER97/Cu interface. Mechanical properties showed a positive correlation with bonding time, as continuous interfaces were achieved for 5 min. However, the noticeable increase in Kirkendall porosity at the EUROFER97/Cu interface in the 9 min sample could contribute to a decrease in shear strength. As validated by microstructural examination, the accumulation of this porosity at the interface could result in the formation of a continuous discontinuity in certain areas. During the application of shear force, the formation and propagation of cracks along this layer are favored, leading to a reduction in the overall strength of the joint.

**Figure 6:**
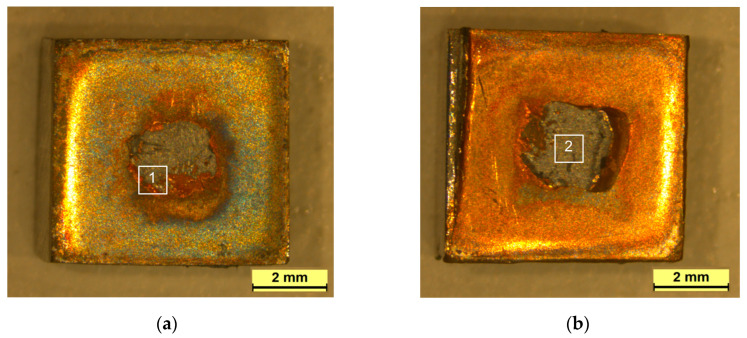

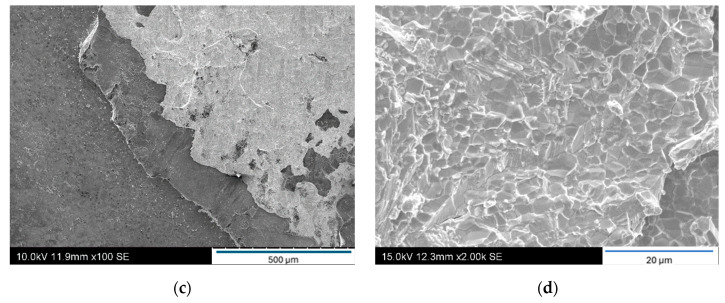
(**a**) EUROFER97 and (**b**) tungsten fracture surfaces of W-EUROFER97 joints using a Cu interlayer after the shear test. (**c**,**d**) detail the zones 1 and 2 marked in figures (**a**,**b**), respectively.

As a consequence of the aforementioned mechanism, shear test failures primarily manifested along the EUROFER97/Cu interface, although it can shift to the W/Cu interface. For instance, Figure 6a,b illustrate the fracture surfaces of W and EUROFER97 components following shear strength tests. In particular, a substantial portion of the remaining interlayer was joined to the W base material, with the exception of the central region of the joint where the interlayer remained in the EUROFER97 base material. The presence of Kirkendall porosity at the EUROFER97/Cu interface elucidates the fracture propagation along this interface.

Utilizing scanning electron microscopy, the highlighted region in Figure 6a was examined, revealing the transition of failure from one interface to another. The contrasting material in the backscattering image of Figure 6c signifies W detachment from the base material during the fracture process, indicating the establishment of a strong interface between the interlayer and W base material. Consequently, when the fracture mechanism shifted from the EUROFER97/Cu to the W/Cu interface, the fracture did not propagate through the interface but rather through the W bulk material. Analysis of the W fracture surface unveiled an intergranular fracture pattern in the W substrate (Figure 6d).

The shear test results presented herein have been compared with those obtained through alternative joining methods utilizing the same Cu interlayer by our research group. Higher shear strengths were achieved through brazing and hot isostatic pressing (HIP) techniques [33,36,37]. Specifically, brazing at 1135 °C for 10 min yielded a shear strength of approximately 220 MPa. It is worth noting, however, that the molten Cu extensively infiltrated the grain boundaries of the EUROFER97 during brazing, leading to significant modifications in the microstructure of the steel near the brazed joint.

Conversely, the results obtained from HIP demonstrated joints with a superior shear strength of approximately 350 MPa, accompanied by minimal alteration in the microstructure of the steel. This outcome was achieved with an extended bonding time of 3 h.

**Figure 7:**
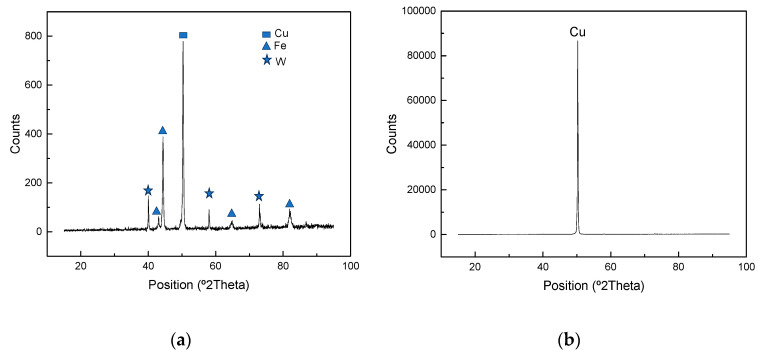
X-ray diffraction patterns of (**a**) EURFOER97 and (**b**) W fracture surfaces.

After the shear test fracture, surfaces on both sides of the joint were analyzed by XRD to determine the crystalline structure and identify the phases that constitute the joint. Figure 7 shows the diffraction pattern, where the presence of Cu, Fe, and W has been identified on the ERUOFER97 side (Figure 7a) and only Cu on the W side (Figure 7b). This information confirms the presence of detached W from the base material, which remained adhered on the EUROFER97 side. The identification of Cu in the W side indicated that almost all copper remained on the W side, as previously indicated. This mechanical behavior has been previously observed in other works when high adhesion properties is achieved between the filler and W base material [38]. No further compounds, such as reaction products or intermetallic phases, have been identified. 

## 4. Conclusions

Tungsten and EUROFER97 were effectively joined using a Cu interlayer material through the field-assisted sintering technique, employing bonding times exceeding 5 min. Shorter durations resulted in discontinuous joints, with only localized points joined at the EUROFERE97/Cu interface. The optimal condition was achieved with a 5 min dwell time at 980 °C. Prolonged dwell times led to the accumulation of Kirkendall porosity at the EUROFER97/Cu interface, giving rise to discontinuous regions in some areas. While the microstructure of the W base material remained unaltered, metallurgical phenomena, such as interdiffusion or dilution, occurred near both interfaces. These phenomena were intensified with longer dwell times at 980 °C.

Bonding time exhibited no discernible influence on the measured microhardness. The bonding process did not affect the hardness of tungsten. However, the EUROFER97 hardness exceeded its as-received condition due to the formation of untempered martensite, necessitating a tempering treatment for restoration.

The maximum shear strength was 120 MPa for the sample joined at 5 min. Higher bonding times increased the interaction at both interfaces, but more importantly, increased the interdiffusion mechanism at the EUROFER97/Cu interface, creating a preferential path for fracture propagation and potentially diminishing shear strength. While failure predominantly occurred at the weaker interface (EUROFER97/Cu), it eventually shifted to the W/Cu interface, causing fracture through the W base material due to the strength of this interface. Consequently, in these regions, detached W remained adhered to the EUROFER97 side of the fracture surface.

## Data Availability

All data are contained within the article.
